# Mean Platelet Volume and Related Parameters May not Contribute to the Diagnosis in Patients with Ascending Thoracic Aortic Aneurysm

**DOI:** 10.21470/1678-9741-2020-0214

**Published:** 2021

**Authors:** Cengiz Beyan, Esin Beyan

**Affiliations:** 1 Retired Professor of Hematology in Ufuk University Faculty of Medicine, Ankara, Turkey.; 2 Department of Internal Medicine, University of Health Sciences, Kecioren Training and Research Hospital, Ankara, Turkey.

Dear Editor,

We read with great interest the retrospective study by Tekin and Tekin related to mean platelet volume (MPV), MPV to platelet count ratio and MPV to lymphocyte ratio in patients with ascending thoracic aortic aneurysm^1^. Based on the results of the study, the researchers suggested that these parameters will contribute to the diagnosis of ascending thoracic aortic aneurysm and will guide the evaluating physician in terms of the need for additional imaging studies. We believe that there are other factors that may have negatively affected and changed the results of this research.

First of all, the fact that the study was designed retrospectively prevented the elimination of pre-analytical and analytical errors, which may have negatively affected the laboratory tests. In addition, the control group did not consist of healthy volunteers, and it was made up of individuals who applied in the hospital at the same time. The fact that the control group is not composed of healthy volunteers and does not represent the society makes it difficult to interpret the results obtained.

As a reason for the research, it has been shown that MPV and its derived parameters were used in diagnosis and prognosis in various diseases. MPV is a complete blood count parameter whose measurement has not been standardized to date and, therefore, it has been reported to have no role in diagnosis and prognosis of acquired diseases^2^. Variables that negatively affect the MPV measurement have been known for a long time, and the main variables are the time from venipuncture to measurement, the anticoagulant used, the method of analysis, the sample storage temperature and the difference in the instruments used in the measurement^3^-^5^. When ethylenediaminetetraacetic acid (EDTA) is present in the blood tube as an anticoagulant, EDTA contact with platelets rapidly causes an increase in MPV. In various studies, the deviation has been reported at a rate of 2-50% in relation to the change in the MPV measurement time^3^. The measurement of MPV with different devices also leads to differences in MPV of up to 40%^4^,^5^. A meta-analysis study involving 181 MPV studies indexed in the PubMed database showed that the MPV measurements could deviate by up to 27.7%, depending on the time variability between venipuncture and measurement and the difference in the measurement devices used^5^. In this study, not knowing the method of MPV measurement raises important concerns about the reliability of the data. Moreover, the fact that it is not known which devices are measured makes the cutoff values defined by the researchers unusable.

As a result, MPV and related parameters may not contribute to the diagnosis in patients with ascending thoracic aortic aneurysm.
